# Soil Water Deficit Reduced Root Hydraulic Conductivity of Common Reed (*Phragmites australis*)

**DOI:** 10.3390/plants12203543

**Published:** 2023-10-12

**Authors:** Ruiqing Wang, Zhenming Zhang, Haoyue Wang, Yinglong Chen, Mingxiang Zhang

**Affiliations:** 1 School of Ecology and Nature Conservation, Beijing Forestry University, Beijing 100083, China; wangruiqing@bjfu.edu.cn (R.W.); haoyuewang@bjfu.edu.cn (H.W.); 2 The Key Laboratory of Ecological Protection in the Yellow River Basin of National Forestry and Grassland Administration, Beijing 100083, China; 3 Wetland Research Centre, Beijing Forestry University, Beijing 100083, China; 4 The UWA Institute of Agriculture, School of Agriculture and Environment, The University of Western Australia, Perth, WA 6001, Australia; yinglong.chen@uwa.edu.au; 5 State Key Laboratory of Efficient Production of Forest Resources, Beijing 100083, China

**Keywords:** root hydraulic conductivity, anatomy, RNA-seq, soil moisture

## Abstract

Alterations in root hydraulics in response to varying moisture conditions remain a subject of debate. In our investigation, we subjected common reeds (*Phragmites australis*) to a 45-day treatment with four distinct soil moisture levels. The findings unveiled that, in response to drought stress, the total root length, surface area, volume, and average diameter exhibited varying degrees of reduction. Anatomically, drought caused a reduction in root diameter (RD), cortex thickness (CT), vessel diameter (VD), and root cross-sectional area (RCA). A decrease in soil moisture significantly reduced both whole- and single-root hydraulic conductivity (*Lp_wr_*, *Lp_sr_*). The total length, surface area, volume, and average diameter of the reed root system were significantly correlated with *Lp_wr_*, while RD, CT, and RCA were significantly correlated with *Lp_sr_*. A decrease in soil moisture content significantly influenced root morphological and anatomical characteristics, which, in turn, altered *Lp_r_*, and the transcriptome results suggest that this may be associated with the variation in the expression of abscisic acid (ABA) and aquaporins (AQPs) genes. Our initial findings address a gap in our understanding of reed hydraulics, offering fresh theoretical insights into how herbaceous plants respond to external stressors.

## 1. Introduction

*Phragmites australis* (Cav.) Trin. ex Steud. (common reed) is a popular perennial grass species that is widely distributed in marsh wetlands throughout the world [1]. This particular species exhibits a broad ecological niche and occupies a wide array of habitats, spanning from freshwater swamps with shallow depths to salt marshes on coastal fronts. Moreover, it assumes a critical function in fulfilling the ecological roles of plant species, participating actively in the material cycling and energy flow processes that characterize wetland ecosystems, and contributing to the stabilization and enhancement of wetland soil structure [2,3]. Global climate changes have become increasingly dramatic, causing extreme erratic weather events to occur more frequently along with longer and/or more intense droughts across the world [4]. Salt marsh wetlands at the interface of the sea and land are among the most fragile ecosystems due to their unique coastal location [5]. Coastal wetlands have been reclaimed for agriculture in many parts of the world, and in China, more than half of the coastal wetlands were once converted to land use [6,7]. As a result of both natural factors and human activities, most of the reclaimed wetlands are located where the tides do not reach, and with insufficient freshwater (precipitation or irrigation) replenishment, wetland plants are at risk of drought [6,8].

In response to alterations in soil environmental conditions, the subterranean root system promptly reacts to soil stress, serving as the foremost physiological and morphological responder [9]. The growth and formation of plant roots under drought conditions is currently a trending research area in plants, such as maize (*Zea mays*) [10], wheat (*Triticum aestivum*) [11], and sorghum (*Sorghum bicolor*) [12], while such studies in wetland plants remain limited. Treating plants at the same phenological stage and duration revealed that moderate drought can stimulate root elongation and downward growth, while severe drought can impede root development, resulting in reduced biomass and root length [13].

The root hydraulic structure is composed of root size and water transport properties, which tandemly affects plant water acquisition capacity under changing or non-uniform soil conditions [14,15]. Root hydraulic conductivity (*Lp_r_*) represents the quotient obtained by dividing the water flux through the root by the disparity in water potential between the root xylem and the soil water potential [16]. It is an important hydraulic parameter for characterizing the capacity of plant water uptake and can be expressed at the whole-root and single-root levels [17]. *Lp_r_* is influenced by multiple root system architecture traits [18], morphological properties [19], and root anatomical properties [20].

In plants, the primary source of hydrodynamic resistance resides within the root system, underscoring the pivotal role of roots in water transport [21]. Root hydraulic resistance consists of radial and axial resistance components [22,23], and axis resistance to water flow is influenced by xylem vessel traits (number, diameter, and area), while in the radial component, it is influenced by cortical traits and the presence of suberized cell layers [24]. Changes in root diameter have been proposed as a trait for increasing plants’ acquisition of water and productivity under drought [25]. Root diameter and tissue density control the length and surface area of root systems for a given biomass allocated to the root system [26], which controls not only the amount of surface directly interacting between the roots and soil, but also the area of root surface colonized by mycorrhizal fungi assisting in plant nutrient acquisition [27]. In addition to root diameter, xylem diameter also affects root hydraulic conductivity and can affect plant productivity under drought [28]. However, to the best of our knowledge, most reports on changes in xylem morphology in response to drought have focused on woody species, and little is known about how the xylem in the roots of herbaceous plants responds to changes in water availability [29].

Numerous studies have shown that abscisic acid (ABA) is a vital regulator of plant drought resistance [30,31]. The hydraulic action of ABA is mediated mainly by the tissue-specific regulation of aquaporins (AQPs) [32]. Several enzymes are involved in ABA synthesis, including 9-cis-epoxide dioxygenase (NCED), cytoplasmic short-chain dehydrogenase/reductase, and aldehyde oxidase (AAO). When soil moisture decreases, roots sense this change and produce ABA in both the roots and leaves through a series of physical and chemical signals, and this induces stomatal closure [33]. An increase in xylem sap pH during drought conditions could potentially enhance the effect of ABA on stomatal closure in plants. Furthermore, the increase in xylem sap pH alone could act as a signal from the roots to the leaves, inducing stomatal closure [34]. However, the influence of ABA on *Lp_r_* under water deficit is still a topic of debate among researchers [30,35].

AQPs are essential transmembrane transporters distributed in the plant membrane system, and regulate *Lp_r_* at the cellular level [21,36]. AQPs consists of five subclasses, including plasma membrane intrinsic proteins (PIPs), tonoplast intrinsic proteins (TIPs), small basic intrinsic proteins (SIPs), nodulin 26-like intrinsic proteins (NIPs), and X intrinsic proteins [21]. AQPs form highly selective pores in most root cell types’ plasma and vacuolar membranes, and have been implicated in the process of plant tolerance to water deficit stress [21]. However, AQPs have mainly been investigated in food crops, with few studies reported on their roles in wetland plants.

Currently, common reed is primarily studied under flooding conditions, with most studies focusing on the changes in its aboveground physiological and ecological characteristics. However, research gaps on common reed root responses to soil water conditions still exist [37]. This study aimed to investigate alterations in the water uptake performance, root morphology, and anatomical structure of the common reed root system under different soil moisture contents, and explore the response mechanism of wetland plant roots to soil moisture content based on the activities of ABA and AQPs, as revealed via transcriptome sequencing. Our hypothesis posited that water deficit would diminish the *Lp_r_* of common reed. This effect would be twofold: influenced by root morphology on one hand, and by the decreased expression of ABA and AQPs on the other hand.

## 2. Results

### 2.1. Root Morphology

All measured indicators showed a gradual decline with decreasing soil moisture contents. The total root length in the 60% and 40% groups was notably less than that in the FC group, constituting 51.25% and 49.11% of the FC group, respectively. Conversely, the 80% group exhibited no significant difference from the other three groups. Moreover, the surface area of the 80%, 60%, and 40% groups accounted for 52.02%, 42.7%, and 30.45% of the FC group, respectively. In line with the observed trend in total root length, the volume of the 60% and 40% groups was significantly diminished compared to that of the FC group, measuring 32.08% and 21.51%, respectively. In terms of average root diameter, there was no significant difference between the treatment groups. Furthermore, there was no significant difference between the three water-deficient groups in all root morphological indicators (Table 1).

### 2.2. Root Anatomy

**Figure 1:**
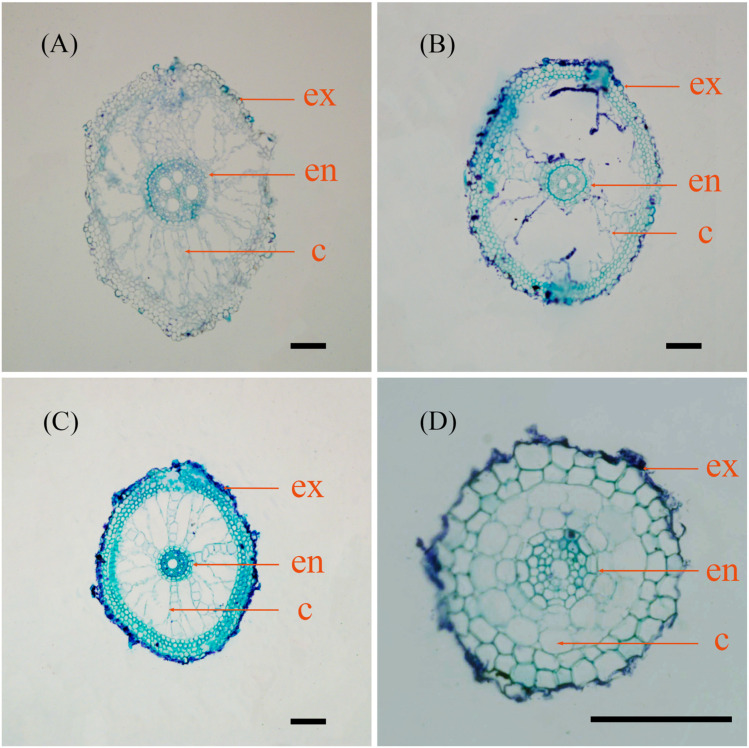
Root cross-section of common reed (*Phragmites australis*) cut at 10 mm from the root tip as observed in (**A**) field capacity treatment, (**B**) 80% field capacity treatment, (**C**) 60% field capacity treatment, and (**D**) 40% field capacity treatment. Bar = 100 μm. ex = exodermis, en = endodermis, c = cortex.

In terms of root anatomical characteristics, we observed inconsistent trends across different parameters. The RD (different from average diameter; the average diameter was calculated by scanning the whole root, and the RD was obtained by measuring the cross section at 10 cm from the root tip) and RCA showed similar morphological trends, decreasing as soil moisture content decreased. In contrast, the FC group exhibited significantly larger root anatomical traits compared to the other three groups. Furthermore, the 80% group exhibited significantly larger root anatomical traits in comparison to the 40% group, with the exception of VD, where the opposite outcome was observed. These findings indicate that the FC and 40% treatment groups had the highest and lowest CT values, respectively, and that there were no significant differences observed when compared to the 80% and 60% treatment groups. Furthermore, the FC and 40% treatment groups exhibited the thickest and thinnest roots, respectively (Table 2). In the FC, 80%, and 60% groups, we observed the presence of aerenchyma contributing to respiration in the root cortex, along with the observation of thickened Casparian bands in the inner cortex. However, no formation of aerenchyma was observed in the 40% treatment group. These results show that the 80% group had the highest average value of vessels, while the 40% group had the lowest (2.44 vs. 1.89). And the average values of the vessels in the FC and 60% groups were 2.11 and 2, respectively (Figure 1).

### 2.3. Hydraulic Conductivity

Except for the 60% group, the value of *Lp_wr_* exhibited an overall decreasing trend with decreasing soil moisture levels (Table 3). Compared to the FC treatment, the *Lp_wr_* of the 80%, 60%, and 40% treatments decreased by 49.39%, 85.98%, and 88.41%, respectively, with the *Lp_wr_* of the FC group being significantly different from those of 60% and 40% treatments. There was no significant difference between the *Lp_sr_* values of all treatment groups.

### 2.4. The Relationship of Lp_wr_ with Root Morphological Features and Anatomical Characteristics

The correlation analysis revealed that the *Lp_wr_* of *P. australis* was significantly positively correlated with the root morphological features, including total root length, root surface area, volume, and average diameter. The *Lp_sr_* was significantly positively correlated with the anatomical characteristics RD, CT, and RCA. Nevertheless, there was no significant correlation between *Lp_sr_* and VD (Table 4).

**Figure 2:**
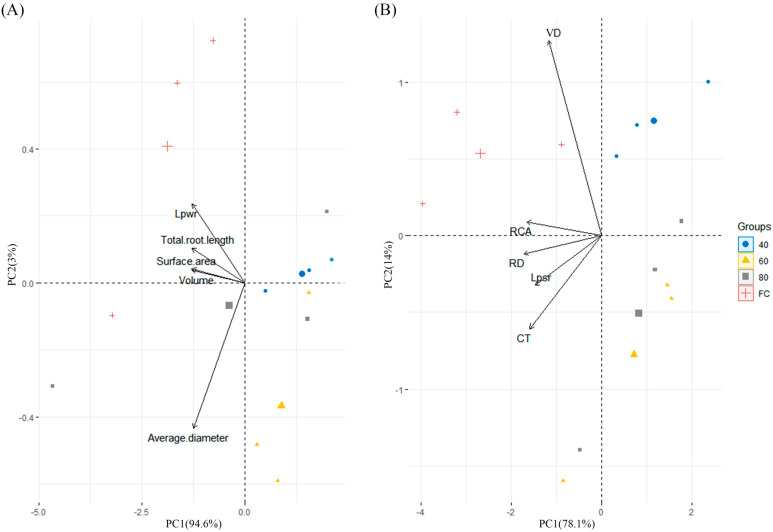
(**A**) PCA of morphological features and the whole-root hydraulic conductivity. (**B**) PCA of anatomical characteristics and the single-root hydraulic conductivity. *Lp_wr_* = whole-root hydraulic conductivity, *Lp_sr_* = single-root hydraulic conductivity, VD = vessel diameter, RCA = root cross-sectional area, RD = root diameter, CT = cortex thickness.

PCA plots of the morphological features and the *Lp_wr_* were depicted, as well as anatomical characteristics and the *Lp_sr_* in Figure 2. The results of PCA indicated distinct clustering patterns among the different watering treatments. The first two principal components (PC1 and PC2) accounted for 97.6% and 92.1% of the total variation observed in the common reed samples, respectively. In Figure 2A, the total root length presented the smallest angle to *Lp_wr_* while the average diameter presented the largest. In Figure 2B, FC group showed a strong tendency with VD, but no covariation of VD and *Lp_sr_* was observed.

### 2.5. RNA-Seq Assembly and Annotation

RNA sequencing analysis showed that the number of clean reads of each transcriptome library ranged between 5.79 and 6.86 Gb, with 90.8%~91.5% of Q30. In addition, the GC of each library was about 53% (Supplementary Table S1). The average length of the expressed genes was 781 with an N50 of 1274. Functional annotation showed that 238,814, 234,312, 147,736, 149,178, and 183,881 expressed genes could annotate to the NR, Swiss-Prot, KEGG, clusters of orthologous genes, and GO database, respectively (Supplementary Table S2). GO analysis revealed that expressed genes related to metabolic processes, cellular processes, and single-organism processes were the most highly represented in the biological process category. Expressed genes related to cells, cell parts, and organelles were highly represented in the cellular component category, while genes related to binding and catalytic activity were highly represented in the molecular function category (Supplementary Figure S1). KEGG enrichment revealed that the expressed genes were involved in five pathways: “Metabolism”, “Genetic Information Processing”, “Environmental Information Processing”, “Cellular Processes”, and “Organismal Systems” (Supplementary Figure S2). Notably, the expressed genes involved in translation were the most represented.

### 2.6. Analysis of DEGs

The analysis of DEGs revealed a total of 886 genes (Supplementary Figure S3). More genes were down-regulated than up-regulated in the 40% and 80% treatments, while the opposite result was observed for the 60% group (Supplementary Table S3). GO functional annotation showed that DEGs were mainly concentrated in antiporter activity, the oxidation-reduction process, and motile cilia (Supplementary Figure S4). KEGG pathway enrichment analysis showed that DEGs were mainly classified as being involved in other types of process, including O-glycan biosynthesis, SNARE interactions in vesicular transport, and starch and sucrose metabolism (Supplementary Figure S5).

### 2.7. The Expression Levels of ABA and AQP Genes and Their Relationship with Lp_r_

**Figure 3:**
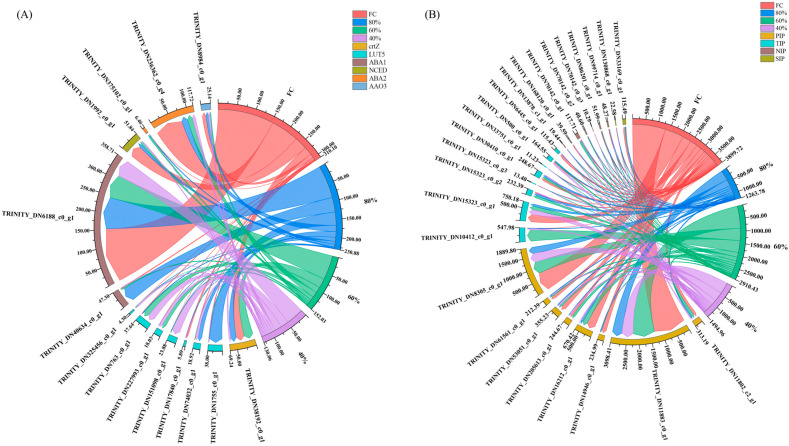
(**A**) Chordal graph showing FPKM expression of ABA DEGs in the FC, 80%, 60%, and 40% treatment groups. FC= field capacity, crtZ = β-Carotene Hydroxylase gene, LUT5 = beta-carotene hydroxylase, ABA1 = zeaxanthin epoxidase (ZEP), NCED = 9-cis-epoxycarotenoid dioxygenase, ABA2 = NAD(P)-binding Rossmann-fold superfamily protein, AAO3 = abscisic-aldehyde oxidase 3. (**B**) Chordal graph showing FPKM expression of AQP DEGs in the FC, 80%, 60%, and 40% treatment groups. FC= field capacity, PIP= plasma membrane intrinsic protein, TIP = tonoplast intrinsic protein, SIP = small basic intrinsic protein, NIP = nodulin 26-like intrinsic protein.

According to the introduction of the importance of ABA and AQPs in plant water transport during drought periods, we screened the gene expression levels of ABA and AQPs via transcriptome analysis, as shown in Figure 3. Genes related to ABA included crtZ, LUT5, ABA1, NCED, ABA2, and AAO3. The total gene expression level of ABA decreased with the decrease in soil moisture. In addition, TRINITY_DN6188_c0_g1 controlling ABA1, TRINITY_DN1992_c0_g1 controlling NCED, and TRINITY_DN236362_c0_g4 controlling ABA2 also decreased with the decrease in soil moisture (Figure 3A). Based on the annotation results, we screened the DEGs of water channel proteins, including PIPs, TIPs, NIPs, and SIPs, and found that they had the highest total expression level in the FC group, followed by the 60% group, and the lowest level was found in the 80% group. Among them, the PIP water channel protein family, which plays a critical role in water transport, had the highest expression levels in the FC and 60% groups (Figure 3B).

**Figure 4:**
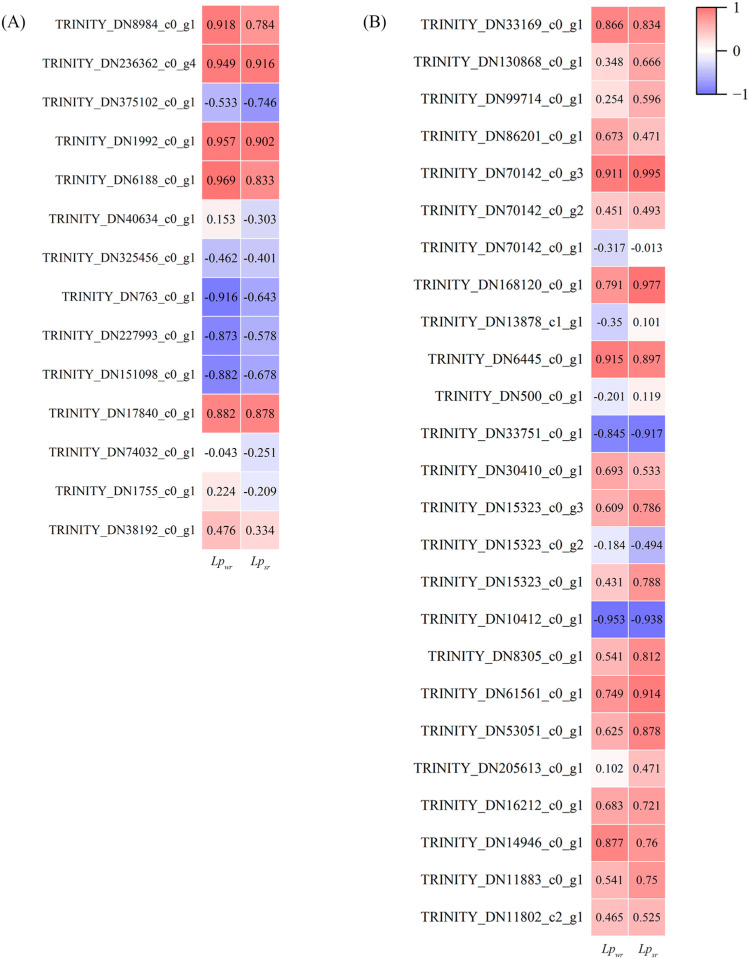
(**A**) Correlation analysis of ABA gene expression and *Lp_r_*. (**B**) Correlation analysis of AQP gene expression and *Lp_r_*. *Lp_wr_* = whole-root hydraulic conductivity, *Lp_sr_* = single-root hydraulic conductivity.

We conducted Pearson correlation analysis between the screened gene expression levels and *Lp_wr_* and *Lp_sr_* (Figure 4). Among the ABA-related genes, TRINITY_DN6188_c0_g1 controlling ABA1 and TRINITY_DN1992_c0_g1 controlling NCED were positively correlated with *Lp_wr_* (*p* < 0.05). Among the AQPs-related genes, TRINITY_DN10412_c0_g1 controlling the TIP water channel protein family was significantly negatively correlated with *Lp_wr_* (*p* < 0.05), while TRINITY_DN168120_c0_g1 and TRINITY_DN70142_c0_g3 controlling NIP were significantly positively correlated with *Lp_sr_* (*p* < 0.05).

## 3. Discussion

As an important water-absorbing and metabolizing plant organ, the plant root system has a direct influence on the growth of plants under environmental stresses [15]. Root growth and hydraulic responses exhibit remarkable plasticity, continuously adapting to numerous soil signals that may have either antagonistic or synergistic effects [22,23]. Changing the root architecture to adapt to different environmental conditions is an important plant coping strategy to stress [38,39]. This study observed the changes in the plant root system of common reed under different soil moisture contents. The results indicate that root surface area, total root length, and root volume of reed plants decreased gradually as water deficit conditions intensified, indicating that drought stress inhibits the growth of root systems. Prior research has demonstrated that early mild drought conditions can stimulate plant root growth. In our study, 80% of the experimental group exhibited some degree of root decline; however, most of the indicators did not reach statistical significance. This observation could potentially be attributed to varietal differences and treatment duration [13]. Plant roots with smaller diameter and higher root length can increase the root surface area in contact with water, which increases the volume of soil available for finding water [40,41]. Simultaneously, they can enhance their *Lp_r_* by diminishing the apoplastic resistance to water entry into the xylem [27]. This is achieved through the promotion of endodermis and exodermis development, the deposition of lignin and suberin, and by relocating the Casparian band closer to the root apices, thus serving as a water-conservation mechanism [42]. Consequently, the plant can acquire the maximum amount of water [43], ultimately enhancing plant productivity during drought stress [25]. For reeds, which do not have longer roots under drought conditions, this may be due to increased mechanical resistance in the soil preventing root elongation [23]. 

A plant’s root anatomy is closely related to its physiological function, which shows how its root system grows. By producing secondary post-growth xylem elements during secondary growth, axial water transport is enhanced, and axial conductance efficiency increases with VD [44]. Generally, a larger VD show greater *Lp_r_*, but also leads to a higher risk of embolism and cavitation [45,46]. In addition, aerenchyma in the root cortex is formed constitutively as well as in response to a variety of stimuli, such as hypoxia, excess temperature, and mechanical resistance [47,48]. In this study, aerenchyma development was also impaired under drought conditions (Figure 1), which may be due to differences in root growth rates and may be more relevant to feeding under flood conditions than to water uptake [49]. Root morphological and anatomical responses to drought conditions likely vary with different plant species or different genotypes of the same species and the extent of drought stress [50].

Previous studies have suggested that *Lp_r_* is regulated by root length, root system architecture, and the distribution of lateral and axial guides [51,52]. *Lp_wr_* was shown to be highest when the soil was hydrated, but decreased with drought intensity [15], which is consistent with our results. Plants with smaller diameters and larger root lengths can increase the surface area in contact with the soil, which, in turn, increases the volume of water absorbed by the root system and decrease the apoplastic barrier, leading to increased *Lp_wr_* [27]. Nevertheless, during natural drought conditions, elongated roots enhance the plant’s ability to access deeper water sources [14]. Furthermore, root hydraulics is more important for the maintenance of root uptake capacity than a lush root system [53].

The *Lp_sr_* is divided into radial and axial conductivity [54], the former reflects the ability of the root to absorb water and the resistance to water movement across the root cortical layers, while the latter indicates the ability of the root to transport water. The root radial and axial *Lp_r_* are expressed by the ratio of the water flux through the whole plant root to the water potential difference between the root xylem and root surface soil [51]. In axial root transport, the primary determining factors are the area and number of vessels. In contrast, Casparian band formation, embolization, root cortex senescence, and the expression of AQPs collectively influence radial water conductivity. Casparian bands impede the flow of substances via the apoplastic pathway between the cortex and vascular cylinder, consequently diverting water and solutions to traverse the cortex through the transmembrane pathway [42,55]. The observed root anatomical structure of other herbs was previously shown to greatly influence *Lp_r_*, while their finer RD was correlated with thinner cortical thickness and higher *Lp_sr_*, and the effects of their CT was greater than those of RD [56]. Higher *Lp_r_* in a small-rooted maize genotype with thinner roots improved water-use efficiency and grain yield compared to a large-rooted genotype under both well-watered and water-stressed conditions [57]. In addition, it is possible that the plants increased their water uptake capacity by increasing the surface area of their root systems, even if this was not reflected in the *Lp_sr_* measurements.

In the soil–plant system, water must flow radially along a series of concentric cell layers in the roots to enter the vascular tissue [58], which includes three transport pathways: the apoplastic, symplastic, and transcellular pathways. The symplastic and transcellular pathways are difficult to distinguish experimentally and are collectively referred to as the cell-to-cell pathway [16]. Composite transport models can be employed to effectively align the root system structure with water transport properties. These models facilitate the integration of root water transport characteristics with the root system structure, enabling the representation of the root hydraulic framework [14]. Depending on environmental conditions, each pathway may change its relative contribution to the total water uptake rate or hydraulic conductivity [52,58]. Furthermore, under drought conditions, root hydraulics adjust by switching between the cell-to-cell and apoplastic pathways, depending on the driving force [59]. Therefore, under transpiring conditions (i.e., during normal daytime water supply), the hydrostatic pressure gradient will control the transport of water and solutes, increasing the contribution of the apoplastic pathway. The apoplastic barrier in the endodermal and exodermal cell walls can block this water transport pathway [60]. Under non-transpiring conditions (i.e., under drought stress), the osmotic gradient will control the transport of water and solutes, following the cell-to-cell pathway [59]. Currently, all of these pathways are known to be interconnected and operate in combination along plant tissue. The contribution of each pathway to radial water flow varies with the plant species, root development stage, and properties of the water uptake driving forces (hydrostatic pressure gradient, osmotic pressure gradient). Changes in the apoplastic water flow are due to changes in the properties of the cell wall [56], while changes in the cell-to-cell pathway water flow involve not only plasmodesmata but also aquaporins, and the secretion of aquaporins is also related to ABA regulation [32].

In this study, TRINITY_DN6188_c0_g1, which regulates ABA1, and TRINITY_DN1992_c0_g1, which regulates NCED, showed a positive correlation with *Lp_wr_*, while ABA2 and AAO3, known to contribute to drought tolerance in plants, were down-regulated [50]. We speculate that ABA secretion plays a role in the cell-to-cell pathway of the reed root system and ultimately regulates *Lp_r_* [32]. The boost in *Lp_r_* due to ABA can be explained as a mechanism to enhance water delivery to the shoot, thereby aiding in the maintenance of water flow throughout the plant during periods of soil or atmospheric water shortage [61]. Previous studies have shown an increase in ABA content in the roots of *Arabidopsis* [62] and wheat [11] under drought stress. However, in a drought experiment on *Phaseolus vulgaris* [63], no significant changes in ABA were observed. While it is rare to observe a reduction in ABA content under drought conditions, it is possible that ABA may change its localization from the symplastic to apoplastic zones during drought treatment. If root ABA is not degraded or lost into the soil water, it could be transferred into the xylem vessels for transport to the leaves [34]. Determining a more precise reason may require further research.

In this study, the PIP genes, which constitute the largest plant AQP subfamily, were highly expressed in the FC and 60% treatments, although the correlation between PIP and *Lp_r_* was not significant. Possible explanations based on previous research suggest that when subjected to drought stress at 60% soil water content, plants respond by up-regulating the expression of water channel proteins. However, under severe drought conditions (40%), the expression of these proteins may be down-regulated [64]. However, the variation patterns of TIPs, SIPs, and NIPs are unclear, likely due to the different families of PIP genes and the varied responses between their isoforms at different time points or stress severity levels [65]. Water conductivity in diverse plant species or organs are controlled differently by AQPs [66]. For example, water and salt stress significantly reduced the *Lp_r_* and AQP activity in *Pinus taeda*, while the *Lp_r_* of *Taxodium distichum* showed little variation. Decreased AQP gene expression reduced *Lp_r_*, making the plant susceptible to wilting [67], or acted as compensation for producing larger root systems [68].

This experiment also has certain limitations. The use of the pressure chamber method usually results in smaller *Lp_r_* values than those measured using root pressure probes [69], and there is also the possibility of water transport through aerenchyma tissue that cannot be effectively avoided. Currently, the measurement of root hydraulic conductivity mainly relies on methods such as root pressure chambers, the capillary hydraulic method, and radioactive isotope tracing, which have some limitations, such as low measurement accuracy and long measurement time. In the future, more advanced, accurate, and rapid measurement methods can be explored. 

## 4. Materials and Methods

### 4.1. Plant Materials

One-node rhizome samples of common reeds were collected from the same community of the Yellow River Delta. The plants were cultivated in the Shengfang greenhouse in Beijing, where air temperature ranged from 28 to 35 °C and there was a relative humidity between 40 and 60% at noon. The level of photosynthetically active radiation on the plant surface was 800–1800 μmol photons m^−2^ s^−1^ (Li—250 A photometer, Li—Cor Biosciences, Lincoln, NE, USA). Plant samples were incubated for two months in the greenhouse to initiate adventitious shoot growth at the stem nodes. Next, we transplanted four genetically identical plants, which were considered biological replicates, into PVC pots (upper diameter 23.2 cm, bottom diameter 18.3 cm, height 21.3 cm). Pot substrates were a mixture of riverbank soil, river sand, and peat (at 4:1:1) with a field capacity (FC) of 34–38% cm^3^ cm^−3^, containing nutrients of 2.29 ± 0.04 mg total N g^−1^ and 0.88 ± 0.01 mg total P g^−1^. After ten days of recovery, plants were treated with four gradients of soil moisture content, including 100% FC, 80%, 60%, and 40% FC, hereafter referred to as FC, 80%, 60%, and 40%, respectively. Plants were manually watered in the morning and evening to keep the soil moisture contents at a relatively stable level of weight. Soil moisture contents were measured 10 cm below the soil surface of each pot using a portable soil moisture meter (HydraGO, Stevens, OR, USA). For each treatment, we randomly selected 3–6 different uniformly sized plants to measure morphological traits, anatomical structures, and hydraulic conductivity and perform transcriptome analysis. It is worth noting that 9 root branches from 3 individuals were used to measure root anatomical traits. All plants were harvested starting from 45 days after treatment, and subsequent measurements were carried out gradually.

### 4.2. Measurement of Root Morphological Traits

At harvest, whole plants were washed with sterile water to remove soil. Then, the clean roots were scanned using a flatbed scanner (Epson Expression 100,000 XL, Seiko Epson Co., Nagano, Japan) at 300 dpi. Subsequently, images were analyzed in WinRHIZO (Regent Instruments Inc., Ottawa, Canada) to quantify total root length, surface area, volume, and average diameter.

### 4.3. Measurement of Root Anatomy

Healthy, freshly grown nodal roots from the rhizome were cut off using a sharp blade, and kept in formalin aceto-alcohol (FAA) solution (90 mL 70% ethanol, 5 mL 100% glacial acetic acid, and 5 mL 37% formaldehyde) for a week before paraffin sectioning. The materials were sealed overnight in 70% ethanol prior to dehydration. The materials were soaked in 70% ethanol, 85% ethanol, 95% ethanol, and 100% ethanol for 2 h. The materials were soaked twice in 100% ethanol, the second time for 1.5 h. For transparency, we soaked the materials for 2 h in a 1:1 solution of V ethanol and V xylene, transferred them to a xylene solution for 2 h, and soaked them again for 1.5 h. The materials were transferred to a fresh solution of xylene, an equal volume of paraffin wax was slowly added in several portions, and the materials were placed in a constant-temperature chamber at 62 °C for more than 4 h to allow the xylene to evaporate slowly, after which the materials were transferred to a pure wax solution for 4 h and soaked 3 times. Forceps were then placed in the incubator for preheating and molten hot wax was poured into a mold for embedding. Slicers were then used to cut the wax blocks into 8–10 μm continuous sections from 10 mm from the root tip (LEICA, RM2126RTQ). Paraffin wax was melted at 58–60 °C, and then, embedded. Xylene and toluidine blue at a concentration of 0.075 g/100 mL were used as the transparent and dye agents, respectively. A microscope (Olympus BX51) was used to observe and take photographs of root sections, and DP2-BSW software was used to measure RD, cortex thickness (CT), vessel diameter (VD), and root cross-sectional area (RCA). 

### 4.4. Hydraulic Conductivity of Roots

Whole-root hydraulic conductivity (*Lp_wr_*) and single-root hydraulic conductivity (*Lp_sr_*) describe the water uptake capacity of the root system at two different levels [17,70]. *Lp_wr_* and *Lp_sr_* under hydrostatic gradients were measured using a TP-PW-II Water Potential System (TPYN SciTech, Hangzhou, China) according to the manufacturer’s instructions, and the specific steps were as follows: Pressure chamber method was used with slight modifications from the previously reported protocol [71]. Plant samples were cut freshly from the base, and then, the underground root parts were placed in the pressure chamber for the measurement of *Lp_wr_*. A small beaker filled with water was placed in the chamber beforehand to ensure continued water uptake by the roots. After initiating the experiment, the pressure was slowly increased, and the incision at the base of the stem started exuding sap immediately. The pressure was maintained for 1 min and its value was recorded. Subsequently, the sap was absorbed using a tampon, and its weight before and after adsorption was measured using a 1:10,000 balance, and the difference was considered the plant sap volume. The roots were then removed from the pressure chamber, and their surface area and total root length were measured using a root scanner. A similar procedure was used to measure *Lp_sr_* for each single root, except that the whole plant was replaced with a single adventitious root; the indeterminate roots were selected in the same way as the roots selected for the anatomical structure measurements above. The ratio of sap flux to pressure difference under a given pressure value was used to determine *Lp_wr_* or *Lp_sr_*, as calculated using the formula below:

(1) $${Lp}_{wr}/{Lp}_{sr}=\frac{V}{s\times t\times P}$$

where *Lp_wr_*/*Lp_sr_* is the whole/single-root hydraulic conductivity (m∙s^−1^∙MPa^−1^); V is the volume of the sap (m^3^); s is the surface area of the root (m^2^); t is the exudate collection time (s); and P is the chamber pressure (MPa).

### 4.5. Transcriptome Sequencing, De Novo Assembly, and Functional Annotation

Before the last harvest, three plants were selected from each treatment group randomly. Fresh and tender root tissues were cut off, washed with sterile water and 75% ethanol, chopped at low temperatures, frozen in liquid nitrogen immediately, and stored until use. TRIzol reagent was used to extract total RNA according to the manufacturer’s instructions (Invitrogen, Carlsbad, CA, USA). The purity and concentration of RNA were assessed using a NanoDrop 2000 spectrophotometer (NanoDrop Technologies, Wilmington, DE, USA) and a Bioanalyzer 2100 system (Agilent Technologies, Santa Clara, CA, USA). We used 1.5% agarose gel electrophoresis to assess RNA integrity. For library preparation, exactly 1 g of RNA was used per sample. Briefly, mRNA was purified from the total RNA using poly-T oligo-attached magnetic beads; then, sequencing libraries were generated from the purified mRNA with unique index codes using the VAHTS Universal V6 RNA-seq Library Kit for MGI (Vazyme, Nanjing, China) following the manufacturer’s recommendations. Subsequently, sequencing was performed using an MGI-SEQ 2000 platform by Frasergen Bioinformatics Co. Ltd. (Wuhan, China). Differentially expressed genes (DEGs) between treatment groups were evaluated using DESeq2 (Love et al. 2014 [72]). False discovery rate (FDR) was used to identify the threshold of the *p*-value in multiple tests in order to compute their significant differences. As a rule, only genes with |log2 (FoldChange)| ≥ 8 and an FDR significance score (padj) < 10^−8^ were included in the subsequent analysis. BLASTX with a cut-off e-value of 10^−5^ was used to compare DEGs against various databases for functional annotations, including the plant-specific sequences from the non-redundant (NR) database https://www.ncbi.nlm.nih.gov/ (accessed on 25 September 2021) and Swiss-Prot database (Kanehisa et al. 2006 [73]). Gene Ontology (GO) annotation was performed based on the correspondence between genes in the NCBI and their GO terms (Ashburner et al. 2000 [74]). Kyoto Encyclopedia of Genes and Genomes (KEGG) pathway annotation was performed using BLASTx against the plant-specific sequences in the KEGG database [73]. GO and KEGG enrichment were performed using the hypergeometric test as implemented in the phyper function in R, version 4.1.1 (R Core Team, 2021).

### 4.6. Data Analyses

Prior to analysis, the homogeneity of variance and normality of the data were checked. Statistical analysis was conducted using SPSS 26.0 software (IBM SPSS Software, New York, United States). Significant differences between means were tested via one-way analysis of variance (ANOVA; Fisher LSD). Correlation analysis and principal component analysis (PCA) were used to explore the relationship between the morphological and anatomical indices and hydraulic conductivity of the common reed root system. Levene’s Test was employed to assess homogeneity of variance, the Shapiro–Wilk Test was utilized for normality assessment, and Pearson correlation analysis was employed to assess correlations. Figures were plotted using R and Photoshop.

## 5. Conclusions

In conclusion, our study has substantiated the profound impact of soil moisture content on diverse root morphological and anatomical traits, along with the modulation of endogenous ABA and AQP expression in common reeds. These variables, in conjunction, significantly influence *Lp_r_*, aligning with our initial hypothesis. Our research has unveiled pivotal insights into how common reeds respond to water deficit conditions.

Firstly, under conditions of water deficit, we observed a substantial reduction in the *Lp_wr_* of common reeds, indicating a diminished ability for water transport. Interestingly, we noted a distinct pattern in *Lp_sr_*, characterized by an initial decrease followed by a subsequent increase. This suggests a dynamic response of the reed root system to changing moisture levels, where initial adjustments may be followed by adaptive changes to optimize water uptake. Moreover, our investigation revealed that the CT increased the resistance of radial conductivity of the root system, while the VD affected the efficiency of axial conductivity of water uptake by the root system. These observations underscore the intricate interplay between roots’ anatomy and hydraulic properties under different moisture conditions. Furthermore, we established significant correlations between the total length, surface area, volume, and average diameter of the reed root system and *Lp_wr_*, highlighting the importance of root morphology in regulating *Lp_r_*. Additionally, root traits such as RD, CT, and RCA were found to be significantly correlated with *Lp_sr_*, emphasizing their role in influencing radial hydraulic conductivity. In light of our transcriptome data, we identified that the expression levels of ABA and AQP genes in reed roots are responsive to drought stress, suggesting a potential regulatory role in hydraulic conductivity. However, a comprehensive understanding of the intricate gene-regulatory mechanisms involved in plant stress resistance remains elusive. Future research should focus on employing integrated genomic, transcriptomic, proteomic, and metabolomic approaches to delve deeper into the molecular mechanisms governing plant responses to various stressors.

In summary, our study not only addresses the influence of soil moisture on reed hydraulics but also highlights the need for a multidisciplinary approach to unravel the complex gene-regulatory networks involved in plant stress responses. Our findings contribute valuable insights to the field and open avenues for further research aimed at enhancing our understanding of plant adaptation to changing environmental conditions, ultimately benefiting sustainable agriculture and ecosystem management.

## Figures and Tables

**Table 1:** Effects of different soil water contents on root morphological characteristics of common reed (*Phragmites australis*).

Treatment	Total Root Length (cm)	Surface Area (cm^2^)	Volume (cm^3^)	Average Diameter (μm)
FC	415.13 ± 59.56 ^a^	125.24 ± 33.58 ^a^	2.65 ± 0.91 ^a^	1042.57 ± 219.89 ^a^
80%	285.83 ± 83.04 ^ab^	65.15 ± 21.12 ^b^	1.67 ± 0.71 ^ab^	970.37 ± 212.8 ^a^
60%	212.77 ± 49.58 ^b^	53.48 ± 13.68 ^b^	0.85 ± 0.29 ^b^	837.3 ± 166.38 ^a^
40%	203.89 ± 56.57 ^b^	38.14 ± 8.79 ^b^	0.57 ± 0.12 ^b^	627.45 ± 88.39 ^a^

The values represent mean ± standard error, *n* = 6. Different alphabetical letters indicate significant differences between groups at *p* ≤ 0.05. FC = field capacity.

**Table 2:** Effects of different soil water contents on root anatomical characteristics of common reed (*Phragmites australis*).

Treatment	RD (μm)	CT (μm)	VD (μm)	RCA (mm^2^)
FC	808.49 ± 20.61 ^a^	298.15 ± 8.03 ^a^	54.82 ± 2.24 ^a^	0.42 ± 0.03 ^a^
80%	601.51 ± 23.62 ^b^	235.94 ± 14.22 ^b^	28.64 ± 0.94 ^c^	0.24 ± 0.02 ^b^
60%	580.91 ± 35.66 ^bc^	238.28 ± 16.8 ^b^	29.66 ± 2.22 ^c^	0.19 ± 0.02 ^bc^
40%	516.1 ± 13.67 ^c^	178.84 ± 6.21 ^c^	44 ± 2.59 ^b^	0.14 ± 0.01 ^c^

The values represent mean ± standard error, *n* = 9. Different alphabetical letters indicate significant differences between groups at *p* ≤ 0.05. FC = field capacity, RD = root diameter, CT = cortex thickness, VD = vessel diameter, RCA = root cross-sectional area.

**Table 3:** Effects of different water conditions on the root system *Lp_r_* of common reed (*Phragmites australis*).

Root Hydraulics	Soil Moisture Contents	*Lp*_r_ (10^−7^ m∙s^−1^∙MPa^−1^)
*Lp_wr_*	FC	1.64 ± 0.26 ^a^
	80%	0.83 ± 0.71 ^ab^
	60%	0.23 ± 0.08 ^b^
	40%	0.19 ± 0.09 ^b^
*Lp_sr_*	FC	1.8 ± 0.51 ^a^
	80%	0.92 ± 0.38 ^a^
	60%	0.89 ± 0.62 ^a^
	40%	0.96 ± 0.54 ^a^

The values represent mean ± standard error, *n* = 3. Different alphabetical letters indicate significant differences between groups at (*p* ≤ 0.05). *Lp_r_* = root hydraulic conductivity, *Lp_wr_* = whole-root hydraulic conductivity, *Lp_sr_* = single-root hydraulic conductivity.

**Table 4:** Whole-root hydraulic conductivity in relation to root morphological characteristics and single-root hydraulic conductivity in relation to anatomical structures of *Phragmites australis*.

Characteristics	*Lp_wr_*	*Lp_sr_*
Total root length	0.949 **	
Surface area	0.971 **	
Volume	0.952 **	
Average diameter	0.866 **	
Root diameter		0.800 **
Cortex thickness		0.755 **
Vessel diameter		0.462
Root cross-sectional area		0.707 *

The Pearson correlation coefficient is given. * *p* < 0.05 and ** *p* < 0.01. *Lp_wr_* = whole-root hydraulic conductivity, *Lp_sr_* = single-root hydraulic conductivity.

## Data Availability

The RNA-seq data have been deposited in the NCBI Sequence Read Archive (SRA) as BioProject PRJNA1025949. All additional data that corroborate the study’s findings can be found in both the main article and its Supplementary Materials.

## Supplementary Materials

The following supporting information can be downloaded at: https://www.mdpi.com/article/10.3390/plants12203543/s1, Figure S1: GO classification map. After GO annotation of all transcript sequences, the successfully annotated transcripts were classified according to the next level of the three major GO categories (BP, biological process; CC, cellular component; MF, molecular function); Figure S2: KEGG classification map. After KO annotation of the transcripts, they were divided into five branches according to the KEGG metabolic pathway in which they are involved: cellular processes, environmental information processing, genetic information processing, metabolism, and organismal systems; Figure S3: Venn diagram showing overlapping sets of DEGs between FC and the 40%, 60%, and 80%, comparison groups; Figure S4: Scatter plot of differential gene GO enrichment. The vertical axis shows the term name, sorted by Qvalue from smallest to largest, the horizontal axis shows -log10(Qvalue), the size of the dots indicates the number of differentially expressed genes in the term, and the color of the dots corresponds to the different RichFactor ranges. (a) DEGs on the 40% group compared with FC; (b) DEGs on the 60% group compared with FC; (c) DEGs on the 80% group compared with FC; Figure S5: Scatter plot of differential gene KEGG enrichment. The vertical axis represents the pathway name, sorted by Qvalue from smallest to largest, the horizontal axis represents −log10 (Qvalue), the size of the dots indicates the number of differentially expressed genes (DEGs) in the pathway, and the color of the dots corresponds to the different RichFactor ranges. (a) DEGs on the 40% group compared with FC; (b) DEGs on the 60% group compared with FC; (c) DEGs on the 80% compared with FC. Table S1: Sequencing data yield statistics; Table S2: Digital gene expression analysis of transcripts under different levels of water deficit; Table S3: Digital gene expression analysis of transcripts under different levels of water deficit.

## Abbreviations

ABA	abscisic acid
NCED	9-cis-epoxycarotenoid dioxygenase
AAO3	abscisic-aldehyde oxidase 3
AQPs	aquaporins
PIPs	plasma membrane intrinsic proteins
TIPs	tonoplast intrinsic proteins
SIPs	small basic intrinsic proteins
NIPs	nodulin 26-like intrinsic proteins
FC	field capacity
FAA	formalin aceto-alcohol
*Lp_r_*	root hydraulic conductivity
*Lp_wr_*	whole-root hydraulic conductivity
*Lp_sr_*	single-root hydraulic conductivity
RD	root diameter
CT	cortex thickness
VD	vessel diameter
RCA	root cross-sectional area
DEGs	differentially expressed genes
GO	Gene Ontology
KEGG	Kyoto Encyclopedia of Genes and Genomes
