# Supplementary material for: Soil Water Deficit Reduced Root Hydraulic Conductivity of Common Reed (Phragmites australis)
Source: Plants (Basel). 2023 Oct 12;12(20):3543. doi: 10.3390/plants12203543 (PMC10610267; doi:10.3390/plants12203543)
Supplement: Supplementary file 1 [file plants-12-03543-s001.zip › Supplementary table.docx]

**Supplementary Table S1 Sequencing data yield statistics**

| Group | Clean reads pairs | Clean base(bp) | Length | Q20 (%) | Q30 (%) | GC (%) |
| --- | --- | --- | --- | --- | --- | --- |
| S | 21,735,441 | 6,520,632,300 | 150;150 | 97.7;96.1 | 91.0;87.6 | 53.5;53.5 |
| A1 | 20,797,590 | 6,239,277,000 | 150;150 | 97.6;95.6 | 90.8;86.1 | 52.8;52.9 |
| A2 | 24,548,784 | 7,364,635,200 | 150;150 | 97.7;96.0 | 91.5;87.6 | 52.2;52.3 |
| A3 | 20,740,186 | 6,222,055,800 | 150;150 | 97.7;96.0 | 91.0;87.0 | 52.1;52.2 |

**Supplementary Table S2 Digital gene expression** **analysis of transcripts under different levels of water deficit**

| Database | Transcripts (Percentage) |
| --- | --- |
| Total | 489,168(100%) |
| KOG | 149,178(30.50%) |
| KEGG | 147,736(30.20%) |
| NR | 238,814(48.82%) |
| GO | 183,881(37.59%) |
| Swiss-Prot | 234,312(47.90%) |
| Unknown | 217,586(44.48%) |

**Supplementary Table S3 Digital gene expression** **analysis of transcripts under different levels of water deficit**

| Group | Up | Down | Total |
| --- | --- | --- | --- |
| 40% vs FC | 1733 | 4534 | 6267 |
| 60% vs FC | 2630 | 428 | 3058 |
| 80% vs FC | 2492 | 6341 | 8833 |
