# Supplementary figures and images for: Soil Water Deficit Reduced Root Hydraulic Conductivity of Common Reed (Phragmites australis)
Source: Plants (Basel). 2023 Oct 12;12(20):3543. doi: 10.3390/plants12203543 (PMC10610267; doi:10.3390/plants12203543)

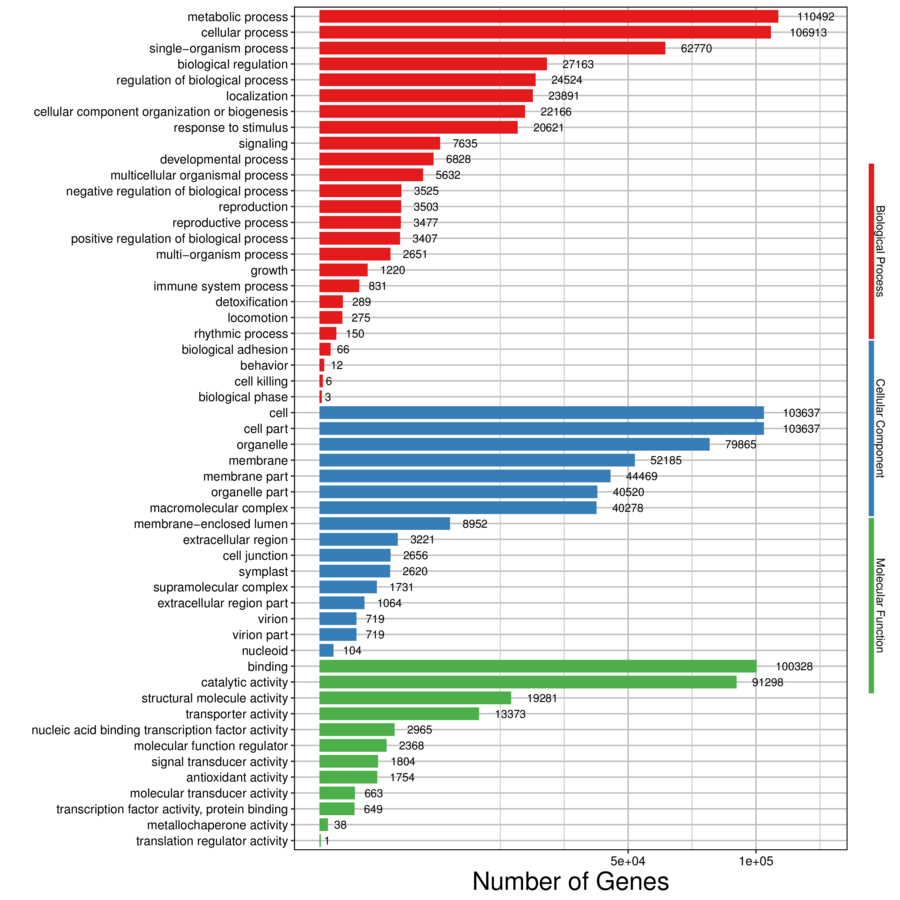

Supplement: Supplementary file 1 [file plants-12-03543-s001.zip › Supplementary Fig. S1.JPEG]

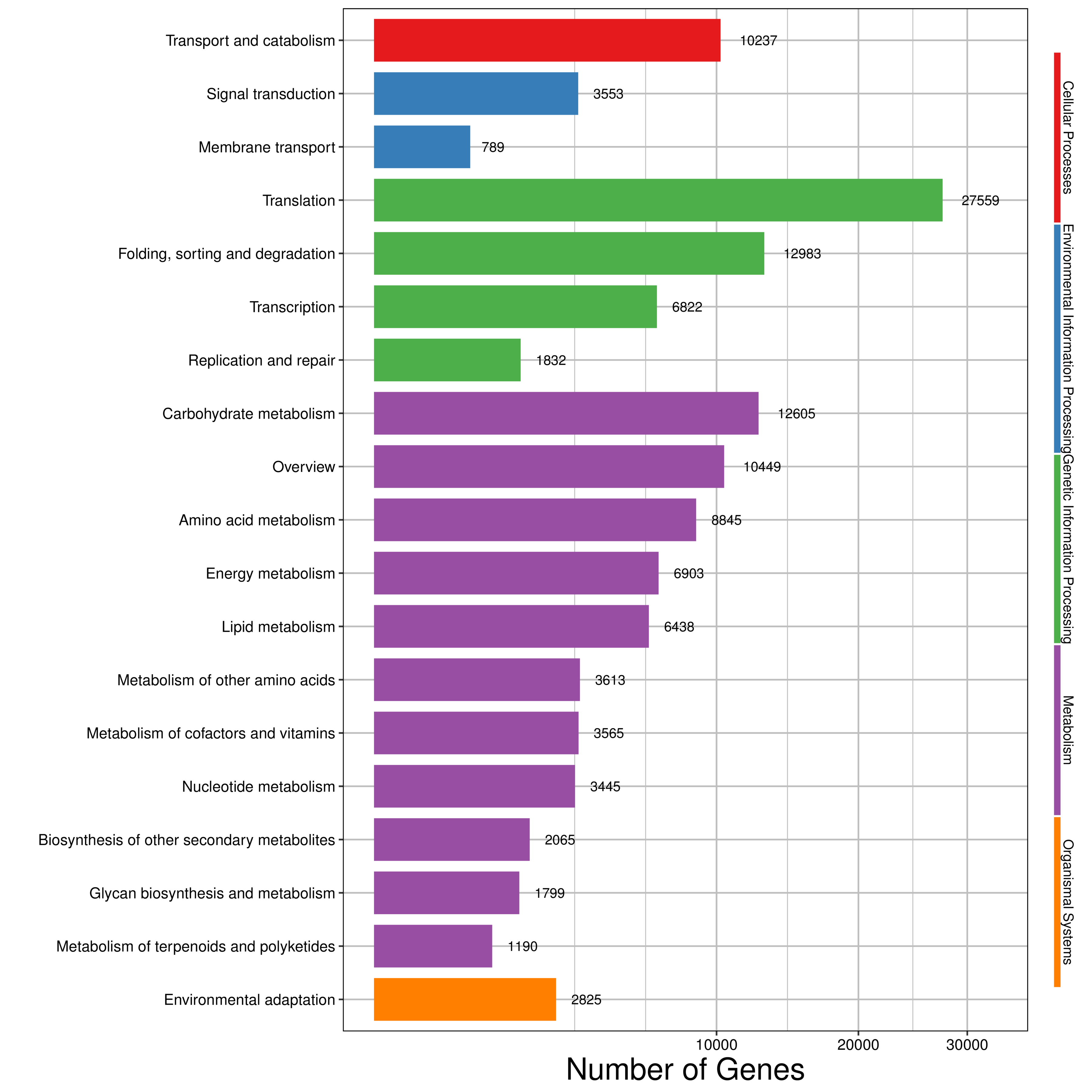

Supplement: Supplementary file 1 [file plants-12-03543-s001.zip › Supplementary Fig. S2.JPEG]

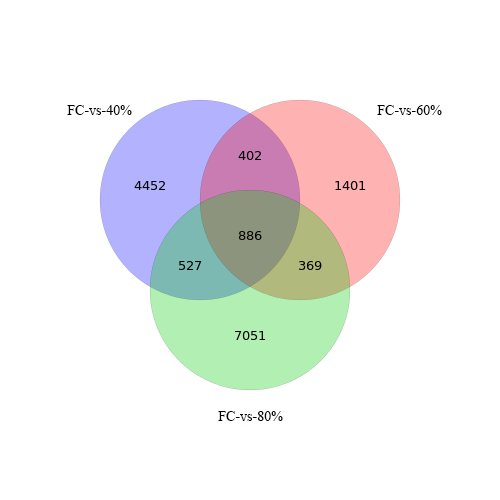

Supplement: Supplementary file 1 [file plants-12-03543-s001.zip › Supplementary Fig. S3.JPEG]

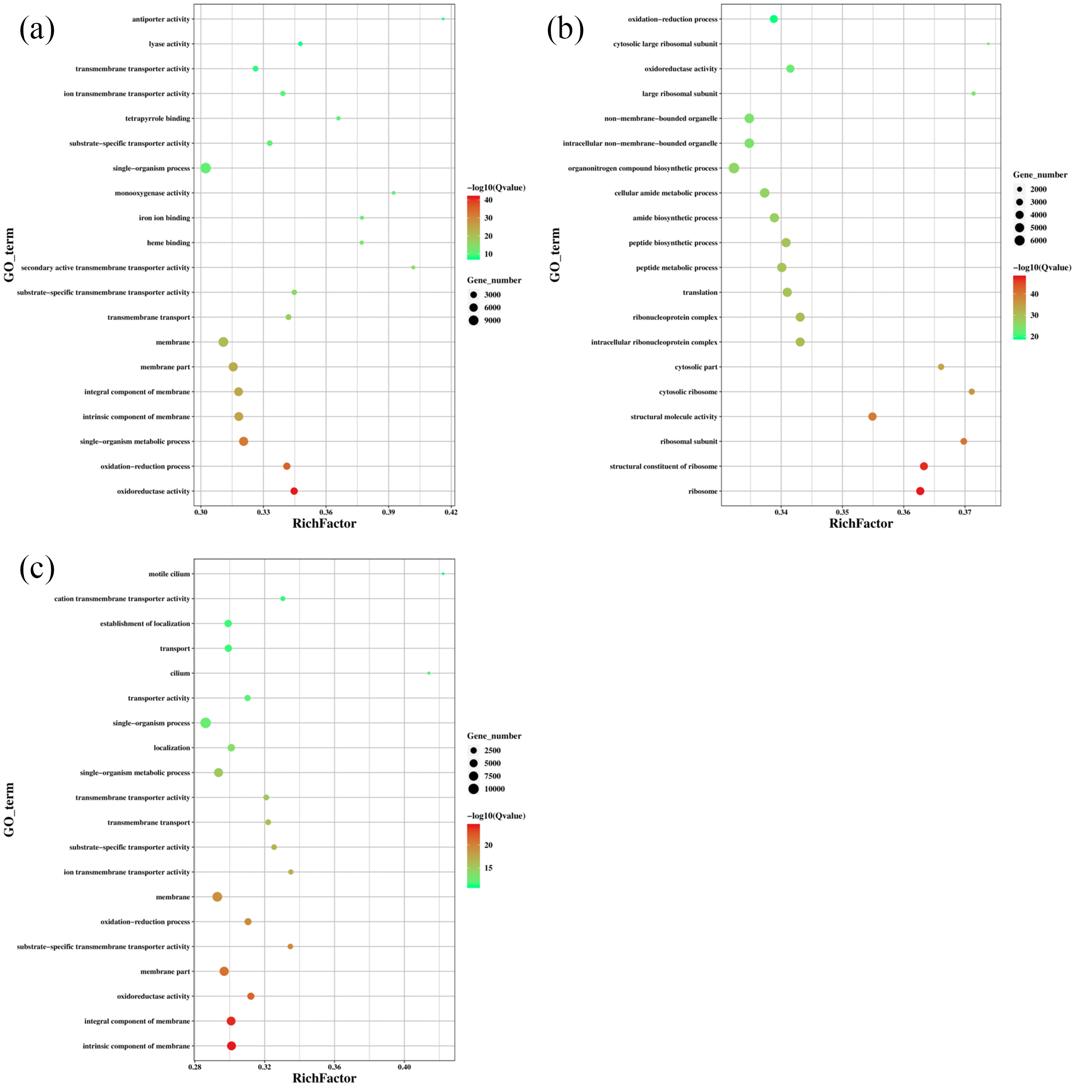

Supplement: Supplementary file 1 [file plants-12-03543-s001.zip › Supplementary Fig. S4.JPEG]

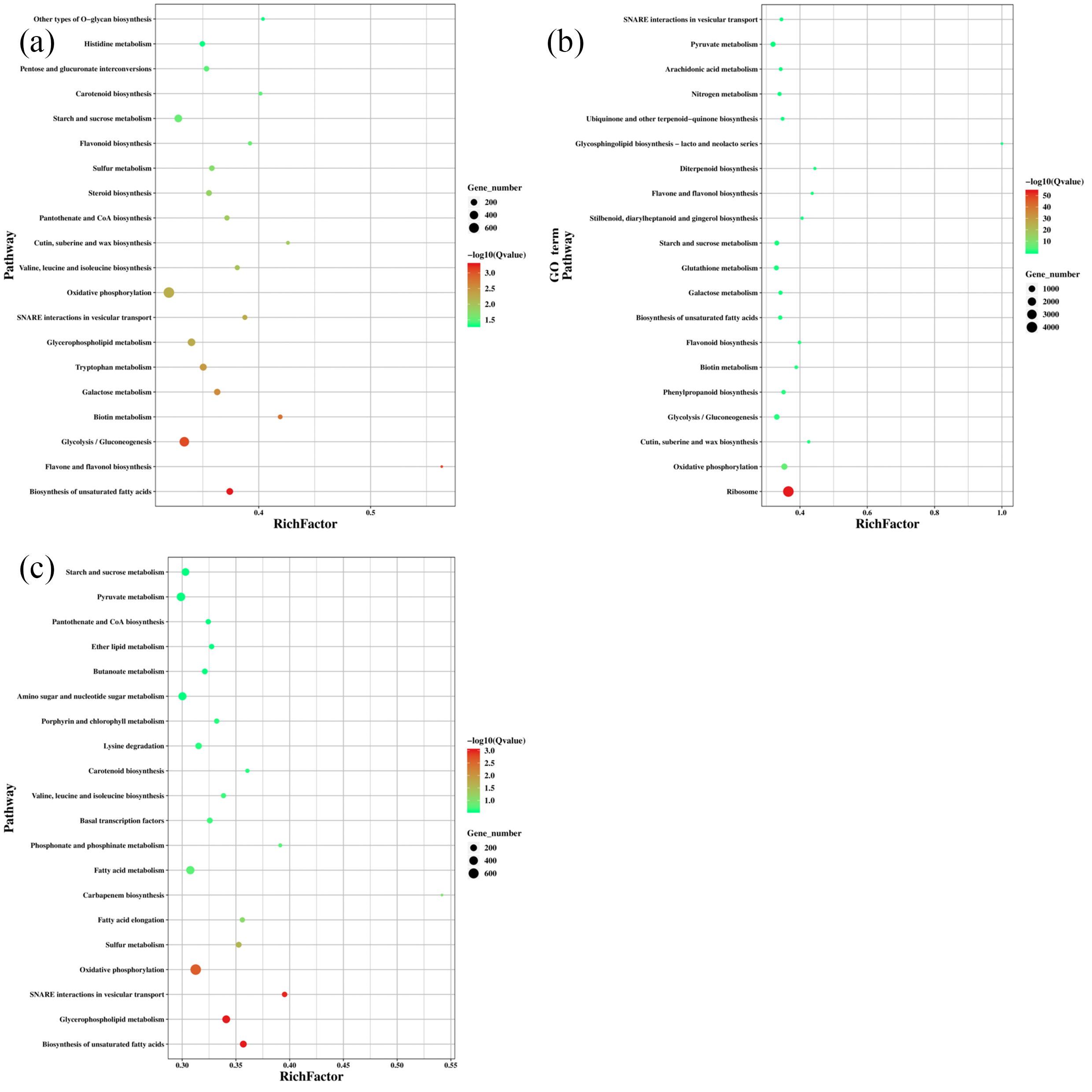

Supplement: Supplementary file 1 [file plants-12-03543-s001.zip › Supplementary Fig. S5.JPEG]
